# A case report of multiple bilateral breast metastases after colorectal cancer

**DOI:** 10.1016/j.ijscr.2021.105759

**Published:** 2021-03-12

**Authors:** Khaled Arnaout, Nouran Hawa, Sarab Agha, Lama Kadoura, Marwa Aloulou, Kusay Ayoub

**Affiliations:** a Faculty of Medicine, University of Aleppo, Aleppo, Syria; b Department of Pathology, Faculty of Medicine, Aleppo University, Aleppo, Syria; c Department of Surgery, University of Aleppo, Aleppo, Syria; d Instructor at General Surgery Department, Aleppo University Hospital, Aleppo University, Faculty of Medicine, Aleppo, Syria

**Keywords:** Colon cancer, Breast metastasis, Immunohistochemistry, Breast tumor, Case report, Colorectal adenocarcinoma

## Abstract

**Introduction and importance:**

Although primary breast cancer is the most common tumor in women, breast metastases are rare findings. We report the first case in English literature with CRC metastases to the breast and adrenal gland concurrently.

**Case presentation:**

A 42-year-old Caucasian female complained of abdominal pain over the last 3 days. Her history was remarkable for stage 2 colon cancer and she was free of disease for 2 years before the presentation, due to receiving Surgical-chemotherapy. The radiologic investigation showed a well-defined cystic mass in the left adrenal gland and left breast nodule. After adrenalectomy, the diagnosis of the mass was metastatic mucinous adenocarcinoma. During the surgical preparation, bilateral breast lumps were noticed. Histopathology of breast mass showed mucinous adenocarcinoma. Immunohistochemical staining revealed that the neoplastic gland was positive for CDX2, CK20, and P53 mutation and negative for CK7, PR, ER, and HER2. Overall, the diagnosis was metastatic colorectal adenocarcinoma to the breast.

**Clinical discussion:**

Metastatic lesions in the adrenal gland tend to be bilateral with irregular shape. Breast metastases are singular unilateral lesions with predominance in the left breast. Biopsy and immunohistochemistry make the final diagnosis.

The management plan is complex and depends on many factors like the general condition of the patient and the presence of other metastases. However, breast metastases may be a clinical clue to disseminated disease.

**Conclusion:**

Breast metastases should be in the differential diagnosis in patients with a history of colorectal adenocarcinoma, in order to provide the appropriate clinical care.

## Highlights

• CRC metastasizes commonly to the regional lymph nodes, liver, lung, and rarely to breast.
• The radiologic investigations can play a role in the differentiation between the diagnosis of primary breast cancer and metastases.
• Immunohistochemically, the majority of breast adenocarcinomas are: negative for CDX2 and CK20 and positive for CK7, while colorectal adenocarcinomas are: positive for CDX2 and CK20, and negative for ER, PR, HER2, and CK7.
• The management plan of CRC metastases to the breast is complex and requires a multidisciplinary team.

## 1 Introduction

Although primary breast cancer is the most common tumor in women, breast metastases are rare findings, with 400 cases reported in the literature [1,2]. The first case of metastatic colonic adenocarcinoma to the breast was reported by McIntosh in 1974 [3].

Detailed medical history, physical examination, and radiologic investigations may distinguish between the primary and metastatic tumor, but the crucial decision is made by biopsy and immunohistochemistry (IHC) [1]. Making an accurate diagnosis is very important to apply the appropriate management [4].

We report the first case of concurrent CRC metastasis to the breast and adrenal gland in a 42-year-old woman, which was a diagnostic challenge given the limited health resources. This study is registered with the ResearchRegistry and the unique identifying number is researchregistry6606 [5]. This case report has been prepared and reported in line with the SCARE guidelines [6].

## 2 Presentation of case

A 42-year-old Caucasian woman presented to our institute complaining of abdominal pain, which started 3 days ago in the left hypochondrium and spread to the back. It was accompanied by nausea, chills, and loss of appetite. She presented no fever, diarrhea, or constipation. Her past medical history was remarkable for stage Ⅱ colonic mucinous adenocarcinoma and she underwent sigmoid colectomy 2 years ago. She received 12 cycles of chemotherapy consisting of folinic acid, 5 fluorouracil, and oxaliplatin (FOLFOX) and she was disease-free for two years after the surgery. She had neither significant family nor pathological psychosocial history. Drug history was clear except for the previous chemotherapy.

Laboratory tests showed a mildly raised level of carcinoembryonic antigen (CEA) of 6.33 ng/ml and slight anemia with a hemoglobin level of 10.1 g/dl and a hematocrit of 30.7%. Biochemical tests were within normal limits. Abdominal ultrasound (US) showed a 9.3 × 14 cm well-defined cystic mass in the left adrenal gland. In the light of CRC history, we scheduled a contrast chest, abdominal, and pelvic computed tomography (CT) scan, which revealed normal findings except for an eight-mm-sized nodule in the left breast and a well-defined cystic mass in the left adrenal with grade 2 hydronephrosis. Because the patient was symptomatic along with the considerable mass size, the surgeon decided to proceed with adrenalectomy and later will investigate the breast nodule. Unexpectedly, we noted more than one lump in her left and right breast during surgical preparation, but was still the surgeon's decision is adrenalectomy to relieve the mass symptoms and clarify its histopathology. The histopathology and IHC of the adrenal mass revealed mucinous adenocarcinoma with a colonic origin.

**Fig. 1:**
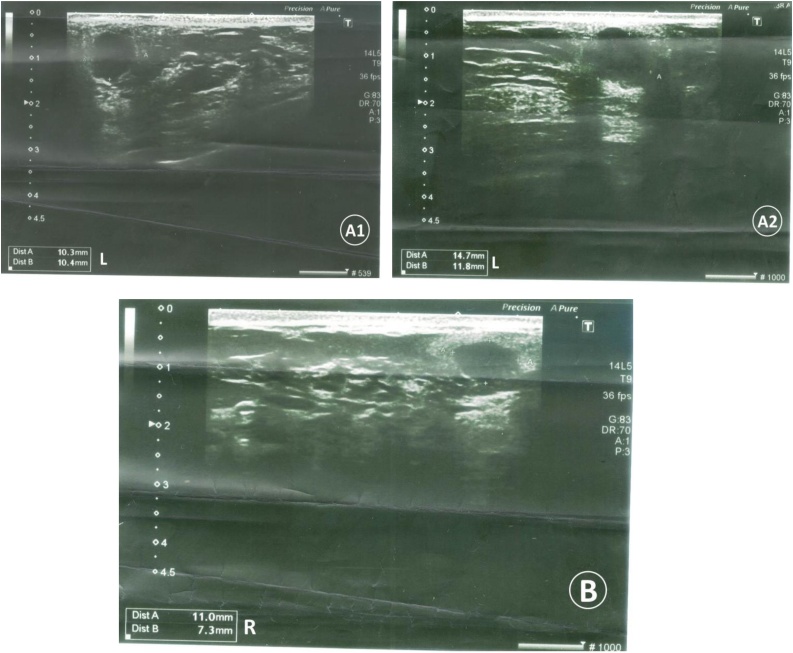
Ultrasound showing (A1, A2) two masses in the left breast (10.3 × 10.4 mm – 14.7 × 11.8 mm) and (B) one in the right breast (11.0 × 7.3 mm) that had irregular margins with central necrotic changes.

**Fig. 2:**
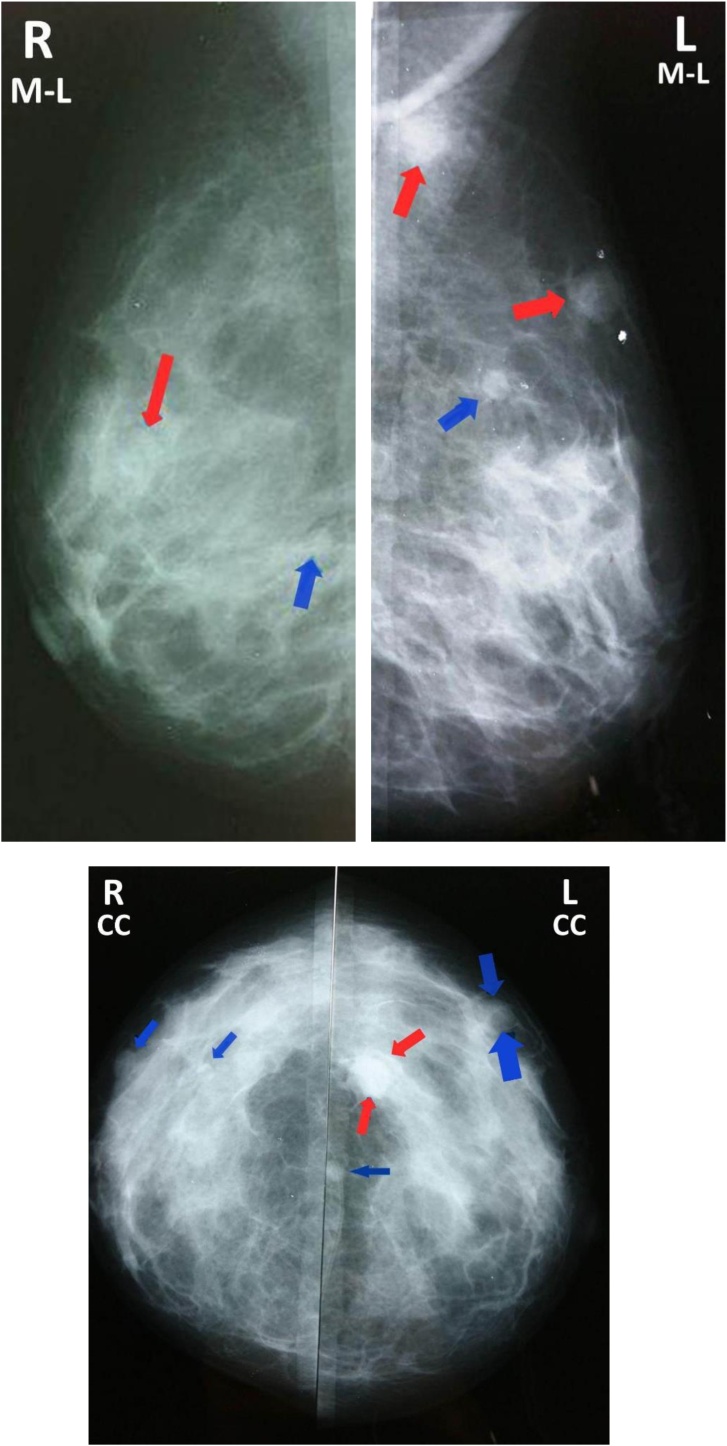
Cranial-caudal and medial-lateral oblique mammographic view showing round multiple bilateral masses (Blue Arrow) and lobulated masses with irregular margins (Red Arrow).

After adrenalectomy, the breast lumps were investigated carefully. On palpation, a lump measuring about 1 cm was felt in the right breast at 9 o’clock position and another three lumps approximately the same size in the left breast at 3 o’clock, 1 o’clock position, and in the axillary tail of the breast. The US showed multiple hypoechoic oval bilateral masses, all of them were well-defined masses except for the one in the right and two in the left that had irregular margins with central necrotic changes (Fig. 1). The mammogram showed multiple rounded masses with no spiculated margins, microcalcification, or thickening of the skin. All masses had regular margins except for one mass in the right breast and two masses in the left breast that were lobulated and had irregular margins (BI-RADS 4A) (Fig. 2). A core needle biopsy was obtained from the largest mass.

**Fig. 3:**
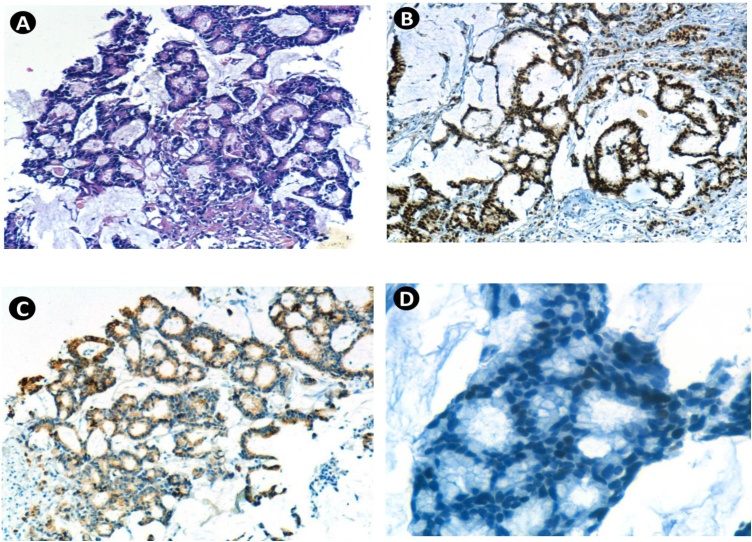
(A) Microscopic section of the tumor showing malignant epithelial proliferation with glandular formations embedded within mucinous lakes and stroma (HE). (B) Positive staining with anti-CDX2. (C) Positive staining with anti-CK20. (D) Weakly and focally positive with anti-P53.

Histopathology showed malignant epithelial proliferation in pools of mucin. Immunohistochemically, the neoplastic glands were positive for caudal-type homeobox transcription factor 2) CDX2), cytokeratin 20 (CK20), and weakly positive for P53 mutation; and negative for cytokeratin 7 (CK7), estrogen receptor (ER), progesterone receptor (PR), and human epidermal growth factor receptor 2 (HER2) (Fig. 3). The final diagnosis was metastatic mucinous adenocarcinoma of the colon.

The tumor tested positive for KRAS mutation with the polymerase chain reaction (PCR) technique. Because the breast metastases were classified as multiple, bilateral and positive for KRAS mutation, the patient was started on FOLFOX chemotherapy without anti-epidermal growth factor receptor (anti-EGFR) drugs for 2 cycles. This showed no improvement so the patient was switched to FOLFIRI chemotherapy consisting of 5-fluorouracil, irinotecan, and leucovorin for 2 cycles, showing no response to treatment.

During chemotherapy, cervical and abdominal US showed multiple lymphadenopathies. After a month of chemotherapy treatments, abdominal US showed two hyperechoic areas in the right liver lobe and mixed echoic mass in the lower part of the abdomen with moderate amounts of ascites. The patient then complained of headaches so a contrast brain CT scan was conducted, and it was normal. The patient was in a poor health condition due to disseminated disease, which progressively deteriorated, and she died two months post adrenalectomy.

## 3 Discussion

Breast cancer is the most common cancer in women. Metastases to the breast are rare, and most of them are contralateral breast cancer, leukemia, melanoma, lymphoma, ovary, lung, and stomach cancer [2,3,7]. CRC metastases to the breast are even more rare, where CRC commonly metastasizes to the regional lymph nodes, liver, and lung [4,8]. CRC metastasis to the adrenal gland is not rare [9], but to our knowledge, this is the first case in English literature with CRC metastases to the breast and adrenal gland concurrently.

Metastatic lesions in the adrenal gland tend to be bilateral and the typical imaging features are irregular-shaped masses. Diagnostic CT-guided fine-needle aspiration (FNA) can help to make an accurate diagnosis when the imaging phenotype is consistent with metastatic disease. However, positron emission tomography (PET) has a high sensitivity for detecting metastatic neoplasms in a patient with a prior history of malignancy when the CT is inconclusive [10].

Our patient posed a diagnostic challenge with our limited resources where the PET scan was not available and the adrenal lesion was a well-defined unilateral cystic mass. The adrenal cyst was differentially diagnosed as a hydatid cyst, as this kind of parasitic infections are common in our region. The FNA in such cysts will cause anaphylaxis shock and the decision with the palliative procedure needs to make the diagnosis of histopathology more accurate. Because the metastases to the breast are rare, the metastases within the breast and the disseminated disease were not considered. Due to the adrenal mass being 15 cm in the greater diameter and symptomatic, we decided to proceed with adrenalectomy.

The median age at which breast metastasis is present is 54 years, and it is found far more frequently in women than in men [4,7,11]. The average interval time between the diagnosis of the breast metastases and the primary source is about 2 years [4]. These findings were consistent with our patient where she had a CRC diagnosis at the age of 40. Then 2 years later, she developed breast metastatic lumps.

The breast lumps diagnosis approach includes a physical examination and radiologic investigations, but the final diagnosis is made by biopsy and IHC.

On physical examination, the CRC metastases to the breast manifest as a readily detectable mobile mass, which does not cause nipple retraction or bloody nipple discharge [1,12]. Breast lesions are characterized as rapidly growing, with the predominance of the left breast, and it is rare to find patients with bilateral or multiple lesions [12]. Our patient’s findings were unusual because she had multiple and bilateral lesions.

The radiologic investigations can play a role in the differentiation between the diagnosis of primary breast cancer and metastases within the breast. US examination of metastatic lesions in the breast shows round or oval hypoechoic masses with microlobulated or circumscribed margins [13]. The classical mammographic findings are round or oval and well-circumscribed masses with no spiculation, microcalcification, or thickening of the skin [13]. Our case had additional characteristics due to necrotic change in the lesions. In the reported case, the mammogram findings were identical to the classical findings except for two masses in the left and one in the right breast that had irregular margins and were lobulated, these characteristics increased our suspicion of primary malignancy; therefore, we did a core needle biopsy to exclude primary breast carcinoma.

Immunohistochemically, the majority of breast adenocarcinomas are negative for CDX2 and CK20 and positive for CK7, while colorectal adenocarcinomas are positive for CDX2 and CK20, and negative for ER, PR, HER2, and CK7 [14,15]. More than half of CRC cases have P53 mutation, but the prognostic significance of this mutation requires further studies for clarification [16,17].

The management plan of CRC metastases to the breast is extremely complex and depends on many factors such as age, clinical characteristics of breast mass, presence of metastases in another site such as liver or lung, and general condition of the patient. According to previous cases, patients with solitary nodules within the breast may benefit from surgical excision with negative margins or excisional biopsy which is usually appropriate for adequate local control especially when the patient is young without comorbidities or other distant metastases [[18], [19], [20], [21]]. Simple mastectomy is sometimes required when the tumor is bulky or painful [3]. Barthelmes et al. [22] recommend that surgical excision should be avoided according to the poor prognosis and the short survival time expectancy and the main type of management for such cases is systemic chemotherapy [23]. 30–50% of CRC have KRAS mutation and have resistance to anti-EGFR agents [24]. The prognosis is poor and the average survival time is considered to be less than one year after diagnosis of breast metastasis [2,3,7].

Our patient started on palliative chemotherapy with no improvement, and died two months after the diagnosis of metastases within the breast. This short survival time is consistent with findings in the literature, and is due to disseminated disease after developing the metastatic disease in the breast.

## 4 Conclusion

CRC metastases to the breast are extremely rare. The appropriate diagnosis requires cooperation between the physician and the pathologist especially given the presence of cancer history. Breast metastases should be in the differential diagnosis for patients with a history of colorectal adenocarcinoma, in order to avoid an unnecessary aggressive procedure. As we noted in this case, that the breast lumps were discovered concurrently with the first CRC metastases, therefore we consider that the breast follow-up in a patient with CRC might be crucial in early diagnosis and efficient management.

## Declaration of Competing Interest

None.

## Funding

This research did not receive any specific grant from funding agencies in the public, commercial, or not-for-profit sectors.

## Ethical approval

Not required for this case report.

## Consent

Written informed consent was obtained from the patient for publication of this case report and accompanying images. A copy of the written consent is available for review by the Editor-in-Chief of this journal on request.

## Authors’ contributions

KA performed the surgery, supervised the study, interpreted the data, and critically revised the article.

SA performed the immunohistochemical staining, interpreted the data, and critically revised the article.

LK, KA, MA, NH made a substantial contribution to conception and design, acquisition of data, analysis, and interpretation of data, drafting, and revision of the article.

All authors read and approved the final version of the manuscript.

## Registration of research studies

researchregistry6606 available at: https://www.researchregistry.com/browse-the-registry#home/registrationdetails/6036d04232cd77001b9733ae/.

## Guarantor

Kusay Ayoub is the guarantor.

## Provenance and peer review

Not commissioned, externally peer-reviewed.

## Availability of data materials

The data are completely available in this article.
